# Promising Anticancer Activity of [Bis(1,8-quinolato)palladium (II)] Alone and in Combination

**DOI:** 10.3390/ijms22168471

**Published:** 2021-08-06

**Authors:** Md Nur Alam, Mohammad Ali Moni, Jun Q. Yu, Philip Beale, Peter Turner, Nick Proschogo, Mohammad Azizur Rahman, M. Pear Hossain, Fazlul Huq

**Affiliations:** 1 Department of Pharmacy, Faculty of Biological Sciences, Jahangirnagar University, Dhaka 1342, Bangladesh; mdnur_kajal@juniv.edu; 2 School of Health and Rehabilitation Sciences, Faculty of Health and Behavioural Sciences, The University of Queensland, St Lucia, QLD 4072, Australia; m.moni@uq.edu.au; 3 Discipline of Pathology, School of Medicine, The University of Sydney, Sydney, NSW 2006, Australia; jun.yu@sydney.edu.au; 4 Sydney Cancer Centre, Concord Hospital, Sydney, NSW 2139, Australia; philip.beale@health.nsw.gov.au; 5 School of Chemistry, The University of Sydney, Sydney, NSW 2006, Australia; p.turner@chem.usyd.edu.au (P.T.); nicholas.proschogo@sydney.edu.au (N.P.); 6 Department of Biochemistry and Molecular Biology, Faculty of Biological Sciences, Jahangirnagar University, Dhaka 1342, Bangladesh; azizurmcdp@gmail.com; 7 Department of Statistics, Bangabandhu Sheikh Mujibur Rahman Science & Technology University, Gopalganj 8100, Bangladesh; mphossain3-c@my.cityu.edu.hk; 8 Department of Biomedical Sciences, City University of Hong Kong, Kowloon 999077, Hong Kong; 9 School of Medical Sciences, The University of Sydney, Kenthurst , NSW 2156, Australia

**Keywords:** cancer, palladium, 8-Hydroxyquinoline, proteomics

## Abstract

Due to similar coordination chemistry of palladium and platinum, a large number of palladium compounds as well have been investigated for their anticancer activity. In the present study, we describe synthesis, characterization, and anticancer activity of palladium complex [Bis(1,8-quinolato)palladium (II)], coded as NH3 against seven different cancer cell lines. NH3 is found to have higher antitumor activity than cisplatin against both parent ovarian A2780 cell line and cisplatin-resistant cell lines. Also, NH3 has the lower IC_50_ value in HT-29 colorectal cancer cell line. The higher antitumor activity of NH3 is due to the presence of bulky 8-Hydroxyquinoline ligand, thus reducing its reactivity. Proteomic study has identified significantly expressed proteins which have been validated through bioinformatics. NH3 has been found to be less toxic than cisplatin at 2.5 mg/kg and 5 mg/kg dosages on mice models. Binary combinations of NH3 with curcumin and epigallocatechin gallate (EGCG) have demonstrated dose and sequence-dependent synergism in ovarian and colorectal cancer models. All of the preclinical studies indicate promising therapeutic potential of NH3 [Bis(1,8-quinolato)palladium (II)] as an anticancer drug.

## 1. Introduction

Serendipitous discovery of cisplatin by Rosenberg in 1970s opened a new horizon in cancer therapy. Since then, several other metal analogues (e.g., complexes of palladium, ruthenium, gold and copper) along with platinum complexes have been investigated in search for better anticancer drugs [1]. Due to similar coordination chemistry of platinum and palladium, new palladium complexes have gained a lot of interest. Although the initial investigations displayed discouraging results marked with greater toxicity and lower anticancer activity for palladium complexes than cisplatin, recently higher antitumor activity in palladium complexes coupled with low toxicity has been achieved by introduction of bulky ligands [2,3].

8-Hydroxyquinoline is a subclass of quinolone family, which has demonstrated a wide variety of biological activities from remote past. 8-Hydroxyquinoline can act as a bidentate chelate that binds with metal ions through the oxygen and nitrogen centers. Many derivatives of 8-Hydroxyquinoline have shown neuroprotection, anticancer, anti-HIV and antifungal actions [4]. In the last few years, a number of research articles from different groups have been published which demonstrate good potential for transition metal complexes containing 8-Hydroxyquinoline derivatives as anticancer agents. Examples include quilamines iron chelator, glycosylated copper(II) ionophores, osmium(VI) nitride complexes, clioquinol copper(II) and zinc(II) complexes, dihalo-8-Hydroxyquinoline-metal complexes, hydroxyquinoline derived vanadium (IV and V), copper(II) and iron(II) complexes, platinum and ruthenium(II) complexes [5,6]. Promising anticancer activity of a platinum complex containing 8-Hydroxyquinoline ligand against ovarian cancer models has also been reported from our laboratory [7]. In the present study, we have described synthesis and characterization of a palladium complex [Bis (1,8 quinolato) palladium (coded as NH3). Anticancer activity of the designed complex against ovarian, colorectal, breast and cervical cancer models has been studied. Proteomic study has been performed to identify the proteins responsible for the anticancer action of the designed complex. Bioinformatics was also implemented to conduct protein–protein interaction, functional enrichment analysis and survival prediction in ovarian cancer using the proteins identified from proteomics. In addition, preliminary toxicity profile of NH3 has been compared with cisplatin in mice model.

Recently combination of chemotherapeutic drugs has demonstrated better outcome in combating cancer and, more specifically, in overcoming drug resistance. Since several phytochemicals have shown good antitumor activity and many of them have already entered into clinic, combination of phytochemicals with other chemotherapeutic drugs might open up new horizon in cancer therapy. Earlier, we have shown synergistic activity from combination of platinum and palladium drugs (cisplatin, oxaliplatin, designed compounds) with phytochemicals against different cancer models [8,9]. In the present study, newly designed complex NH3 has been applied in combination with two naturally derived phytochemicals curcumin and EGCG against ovarian and colorectal cancer models as a function of concentration and sequence of administration.

## 2. Results and Discussion

### 2.1. Synthesis

**Figure 1:**
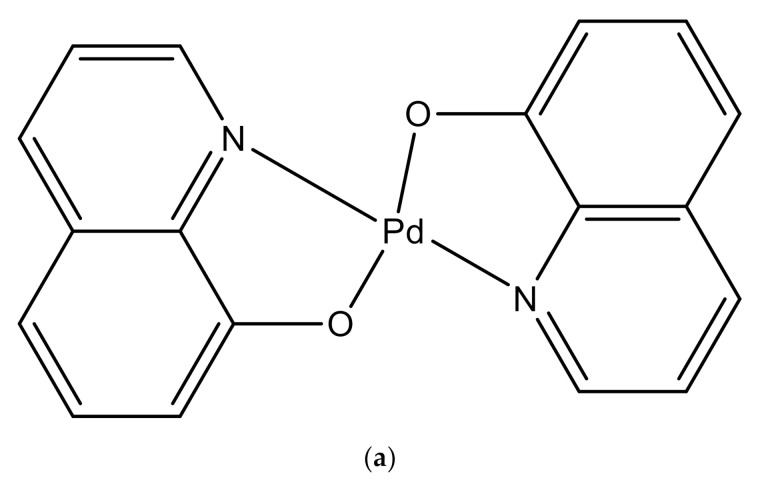

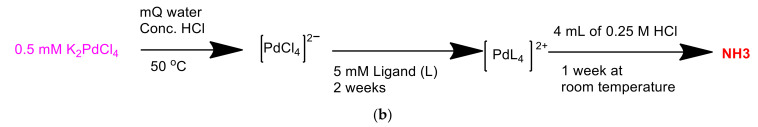
(**a**) Schematic for the structure of the synthesized complex NH3; (**b**) Flow diagram for the synthesis of the compound NH3.

The complex coded as NH3 shown in Figure 1a was synthesized by reacting potassium tetrachloropalladate with 8-Hydroxy quinoline ligand. The synthesis scheme of the complex is shown in Figure 1b. Synthesis of the same complex was described earlier in 1960s, but we have synthesized NH3 using a different method. 

### 2.2. Crystal Structure of NH3

**Figure 2:**
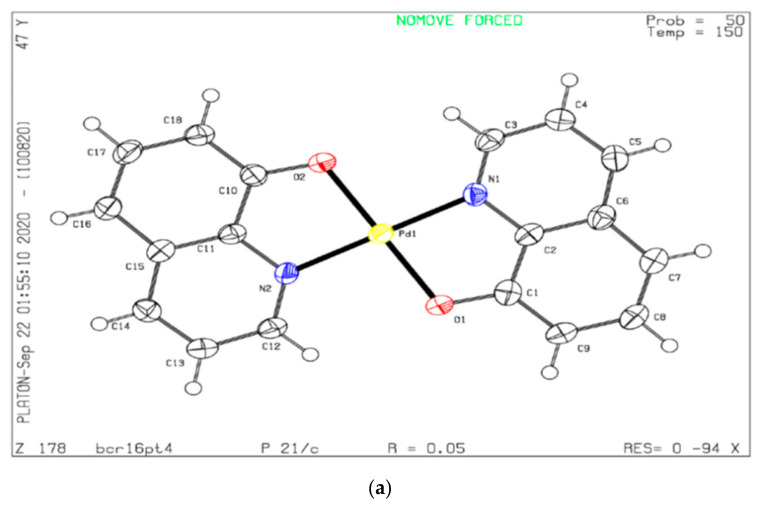

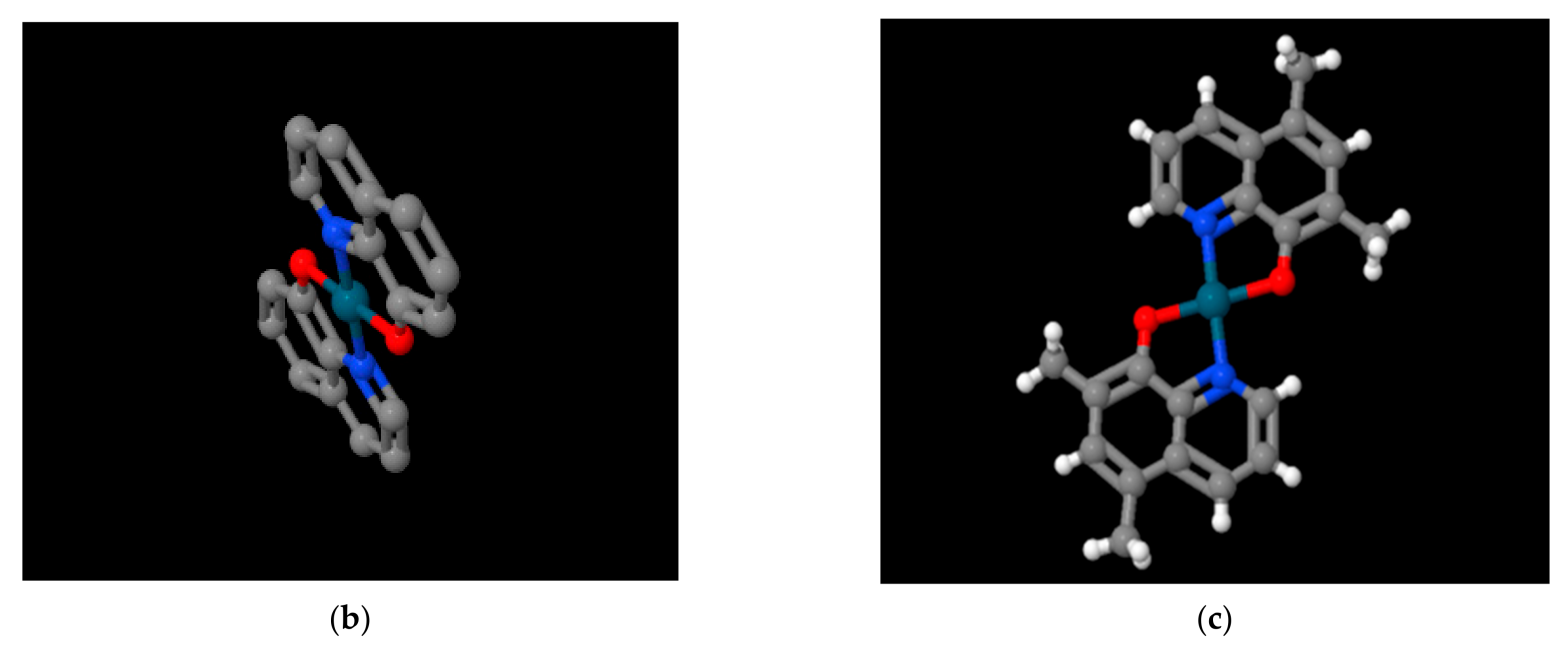
(**a**) ORTEP Structure of NH3 (**b**) ORTEP structure of HQUIPD (**c**) ORTEP structure of HQUIPD01.

Crystallographic details and selected bond lengths and angles for NH3 are provided in Tables S1 to S3 of the Supplemental Information (SI). The square planar coordination geometry of the complex molecule is unremarkable. The CCDC deposition numbers for NH3 is 2033432. Presumably reflecting differences in crystal growth procedures, the crystal structure packing and hence unit cell of NH3 differs from that of the structure reported by Prout and Wheeler [10] in 1966 (CCDC code: HQUIPD) and by Low, Xu, Xiang and Chu [11] in 2011 (CCDC code: HQUIPD01). The 1966 and 2011 structures are respectively reported in the non-standard P2_1_/b and P2_1_/n monoclinic settings, with the latter being an appropriate choice for the 2011 structure in having a beta angle closer to 90 degrees than its P2_1_/c standard setting counterpart. The P2_1_/n setting would likewise be beneficial for the 1966 structure. In contrast, the NH3 structure reported here is best represented in P2_1_/c. Differences in the unit cells of the 1966, 2011 and present structure are evident in Table 1, which lists the unit cell constants of all three in the standard P2_1_/c setting. The differences in the cell constants for the isomorphous 1966 and 2011 structures will at least in part reflect different data collection temperatures. ORTEP structures of NH3, HQUIPD and HQUIPD01 is presented in Figure 2a–c.

A key difference is that in the isomorphous HQUIPD and HQUIPD01 structures, the complex molecule is located on an inversion center, whereas in the NH3 polymorph reported here, the molecule is located on a general position. The metal to metal distance in the HQUIPD01 structure is 4.7187(3) Å, in contrast to 3.7751(5) Å in the NH3 structure presented here. The inter-planar separation between offset-stacked molecules is approximately 3.29 Å in the HQUIPD01 structure and 3.42 Å in the NH3 structure. The stacking lateral offset is then approximately 3.38 Å in the HQUIPD01 structure and 1.60 Å in in the NH3 structure.

### 2.3. Antitumor Activity of the Complex

**Figure 3:**
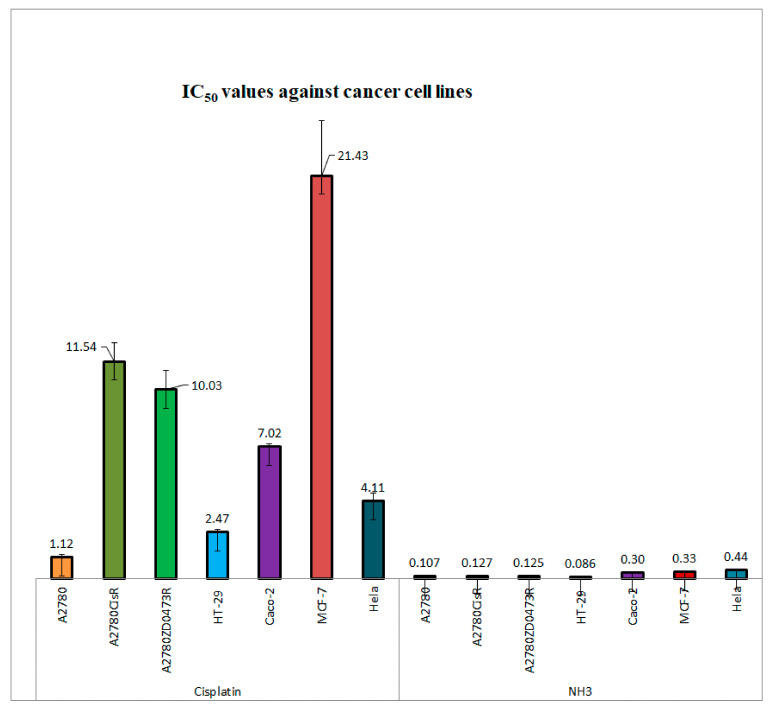
IC_50_ values (µM) against a variety of human cancer cell lines (After 72 h, MTT assay, 3 repeats).

MTT reduction assay was used to determine cytotoxicity of the designed complex NH3 and standard anticancer drug cisplatin against seven (three ovarian i.e., A2780, A2780^cisR^, A2780^ZDO473R^; two colorectal HT-29, Caco-2; one cervical Hela and one breast MCF-7) different cancer cell lines. The results of the comparative antitumor activity between NH3 and cisplatin has been displayed in Figure 3. Dose response curves have been presented in Supplementary File (Figures S1–S5). It can be seen that the newly designed complex NH3 has demonstrated greater anticancer activity than cisplatin against all tested cell lines.

Among the tested cancer cell lines, NH3 showed 10 times greater activity than cisplatin against parent ovarian A2780 cell line. Anticancer activity of NH3 was even greater in cisplatin against the resistant cell lines (90 times more in A2780^cisR^ cell line and 80 times more in A2780^ZDO473R^) compared to cisplatin. The greatest anticancer activity of NH3 in terms of lowest IC_50_ value was observed in HT-29 colorectal cancer cell line. In other cancer models as well NH3 exhibited greater cytotoxicity than cisplatin: 28 times greater in Caco-2 cell line; around 65 times greater in MCF-7 cell line and around 10 times greater in Hela cell line. A number of studies have been conducted earlier towards evaluation of anticancer activity of the ligand 8-Hydroxyquinoline itself and its derivatives. 8-Hydroxyquinoline exhibited significant antitumor activity against Raji (lymphoma), Hela (cervical) and PC-3 (prostate) cancer cell line [12,13]. Few derivatives of 8-Hydroxyquinoline also displayed very high anticancer activity during in vivo and in vitro model study i.e., clioquinol [14], nitroxoline [13] and mannich bases of 8-Hydroxyquinoline [15]. Interestingly, chelation of metals with 8-Hydroxyquinoline or its derivatives further increased antitumor activity. Six copper chelated compounds have been reported to show lower IC_50_ values (1.3–16 µM) against PC-3 and Hela cell lines [12]. Promising antitumor activity of platinum chelated compounds also has been described earlier [5,7]. Palladium compounds containing clioquinol were also reported to possess significant anticancer activity against ovarian cancer model [16]. However, NH3 is found to show superiority (in terms of IC_50_ values) over the all previously reported results on activity of 8-Hydroxyquinoline, derivatives of 8-Hydroxyquinoline, metal chelated 8-Hydroxyquinoline or derivatives against various tumor models. 

The higher antitumor activity of NH3 can be partially attributed to the presence of the ligand 8-Hydroxyquinoline itself. Coordination of the ligand with palladium metal might have resulted in further increase in activity which has been observed in earlier studies with copper and platinum [5,12]. Four-coordinated square planar geometry of NH3 where two 8-Hydroxyquinoline ligands arrange in the trans position with palladium might be in the unique position to bind and DNA and maximize killing of cancer cells.

### 2.4. Proteomics

The study has provided information on the underlying mechanism of antitumor activity of NH3 alone in different cell lines. Total of 19 (seven from A2780 cell line, nine from A2780^cisR^ cell line and three from HT-29 cell line) proteins have been identified from the present study that underwent significant changes in expression following treatment with NH3 alone. Change in folds of the respective proteins after treatment with NH3 alone is shown in Table 2 (A2780 cell line), Table 3 (A2780^cisR^ cell line) and Table 4 (HT-29 cell line).

**Figure 4:**
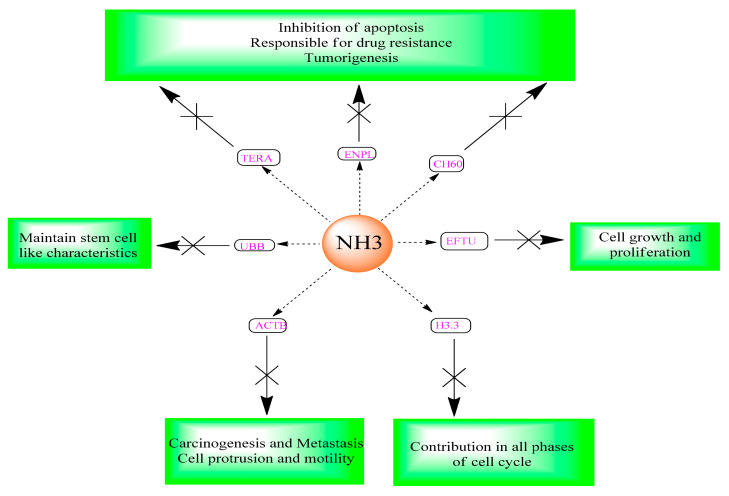
Anticancer mechanism of NH3 obtained from proteomics.

Fourteen proteins including: actin cytoplasmic 1 (ACTB), vimentin (VIME), endoplasmin (ENPL), 60 kDa heat shock protein (CH60), 78 kDa glucose-regulated protein (GRP78), polyubiquitin-B (UBB), histone H3.3(H3.3), cofilin-1 (COF1), heat shock cognate 71 kDa protein (HSP7C), 40S ribosomal protein SA (RSSA), keratin type II cytoskeletal 1 (K2C1), citrate synthase (CISY) elongation factor Tu (EFTU) and transitional endoplasmic reticulum ATPase (TERA) have been downregulated and considered to be associated with the anticancer action of NH3 in ovarian cancer. Downregulation of three proteins namely: nucleoside diphosphate kinase B (NDKB), Annexin A1 (ANXA1) and histone H4 (H4) have been thought to be related to the anticancer action of NH3 in colorectal cancer. Among the above identified proteins, seven of them was considered to be highly significant (more than 3-folds downregulation) in relation to the antitumor action of NH3 (Figure 4).

### 2.5. Protein–Protein Interaction and Functional Enrichment

**Figure 5:**
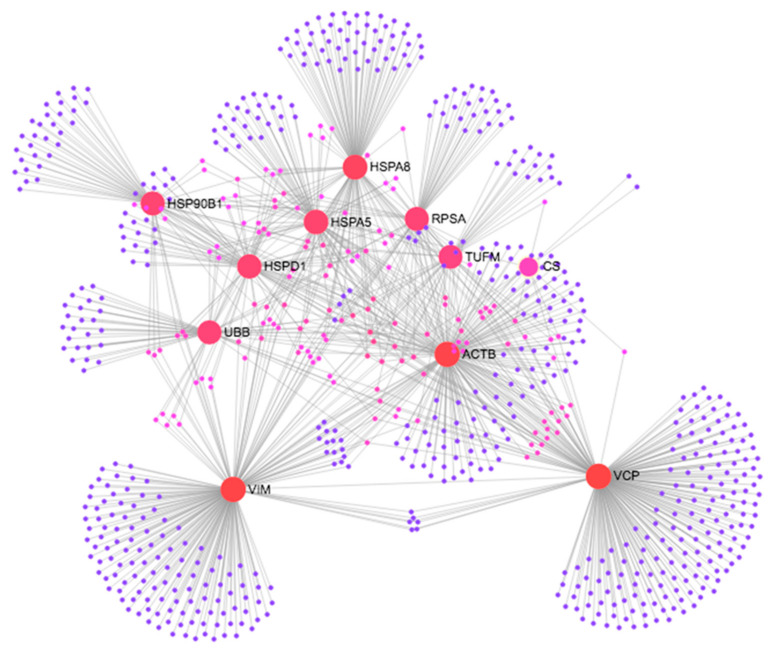
Protein–Protein Interaction of the proteins identified in the ovarian cancer cell line.

We have carried out protein–protein interaction analysis using the proteins identified in ovarian cancer. In the study, we have used protein–protein interaction database “STRING” [17] and NetWorkAnlyst [18] software tools. We found that among the 14 proteins, 11 of them namely: ACTB, HSPD1 (corresponding to CH60), CS (corresponding to CISY), TUFM (corresponding to EFTU), HSP90B1 (corresponding to ENPL), HSPA5 (corresponding to GRP78), HSPA8 (corresponding to HSP7C), RPSA (corresponding to RSSA), VCP (corresponding to TERA), UBB and VIM (corresponding to VIME) are strongly connected together as shown in Figure 5. Hence it appears that these altered proteins bind together with NH3 thus accounting for its antitumor activity.

**Figure 6:**
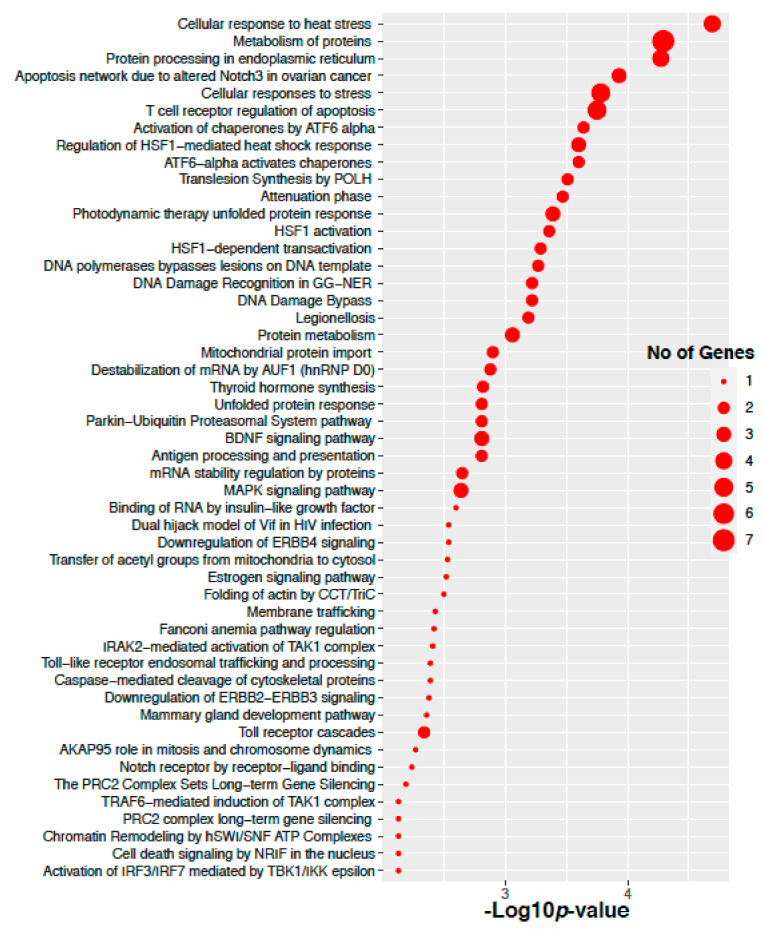
Pathway enrichment of the genes corresponding to the altered 14 proteins in the ovarian cancer.

**Figure 7:**
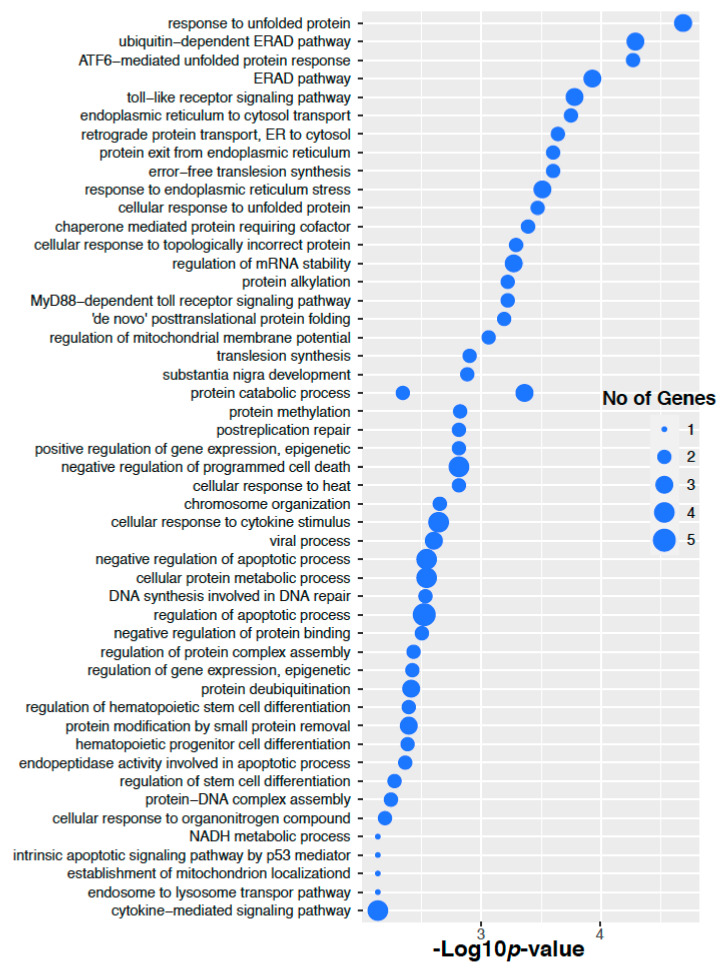
Gene Ontology enrichment of the genes corresponding to the altered 14 proteins in the Ovarian cancer.

We have performed signaling pathways enrichment analysis using EnrichR software tools [19] that incorporated several pathways databases including KEEG, WikiPathways, REACTOME, BioCarta, BioPlanet and Panther. Here we have considered the genes corresponding to the altered 14 proteins in the ovarian cancer. Top 50 significant signaling pathways and their −10 logarithmic *p*-values are shown in Figure 6. We have found several valid significant cancer related Gene Ontology pathways that are associated with these altered 14 proteins in ovarian cancer. We have also performed Gene Ontology pathways enrichment analysis using the Gene Ontology biological process database [20]. Here we have considered the genes corresponding to the altered 14 proteins in the ovarian cancer. Top 50 significant Gene Ontology pathways and there −10 logarithmic *p*-values are shown in Figure 7. We have found that there are several significant Gene Ontology pathways related to cancer included the genes corresponding to the altered 14 proteins in the ovarian cancer.

**Figure 8:**
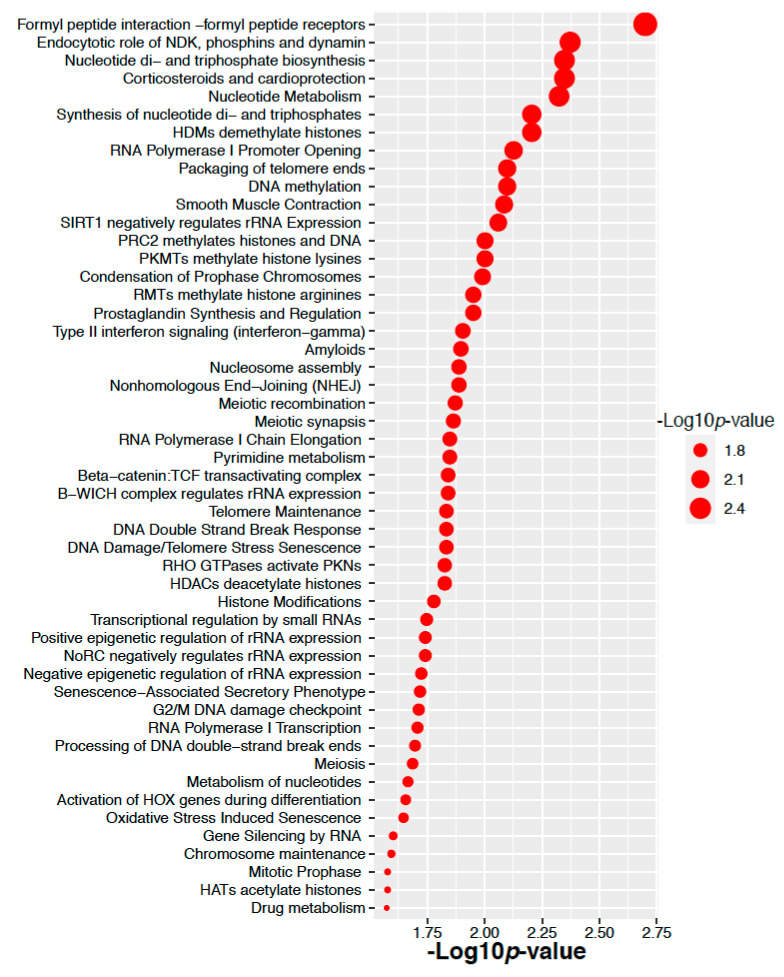
Pathway enrichment of the genes corresponding to the altered 3 proteins in colorectal cancer.

**Figure 9:**
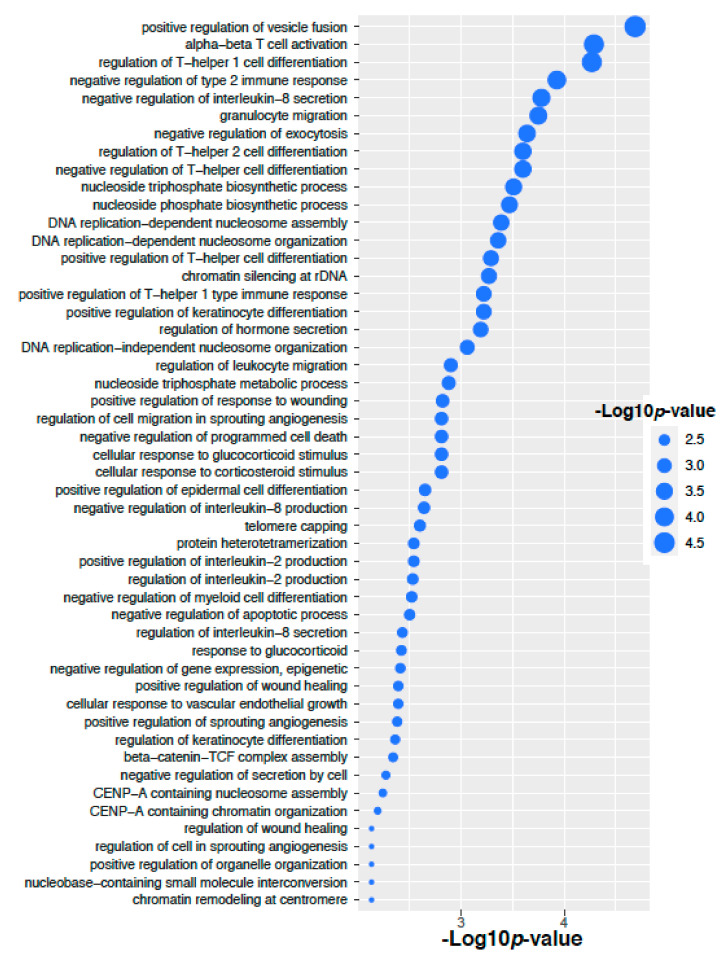
Gene Ontology enrichment of the genes corresponding to the altered 3 proteins in colorectal cancer.

For colorectal cancer, we have considered the genes corresponding to 3 altered proteins in colorectal cancer. Top 50 significant signaling pathways and their −10 logarithmic *p*-values are shown in Figure 8. We found that there are several significant signaling pathways related to the cancer included the genes corresponding to the altered 3 proteins in colorectal cancer. We have also performed the Gene Ontology pathways enrichment analysis using the Gene Ontology biological process database. Here we have considered the genes corresponding to the altered 3 proteins in the ovarian cancer. Top 50 significant Gene Ontology pathways and their −10 logarithmic *p*-values are shown in Figure 9. We found that there are several significant Gene Ontology pathways related to the cancer included the genes.

### 2.6. Survival Prediction of the Ovarian Cancer Proteins

**Figure 10:**
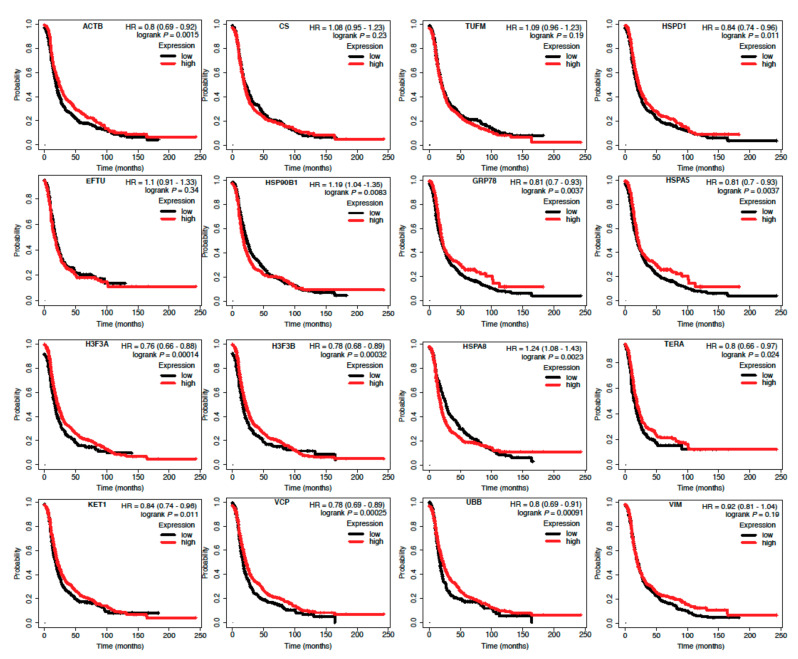
Survival prediction of the 16 genes corresponding to the altered 14 proteins in the ovarian cancer.

We have considered mRNA and clinical data from the TCGA database to predict the survival of the genes corresponding to the altered 14 proteins identified in ovarian cancer [21]. The Kaplan-merrier plots for the 16 genes corresponding to the 14 proteins of the ovarian cancer are shown in Figure 10. We observed that 12 genes (*ACTB*, *HSPD1*, *HSP90B1*, *GRP78*, *HSPA5*, *H3F3A*, *H3F3B*, *HSPA8*, *TERA*, *KET1*, *VCP*, *UBB*) among the 16 genes showed significant effect on the ovarian cancer survival.

### 2.7. Altered Proteins Associated with the Cancer

**Figure 11:**
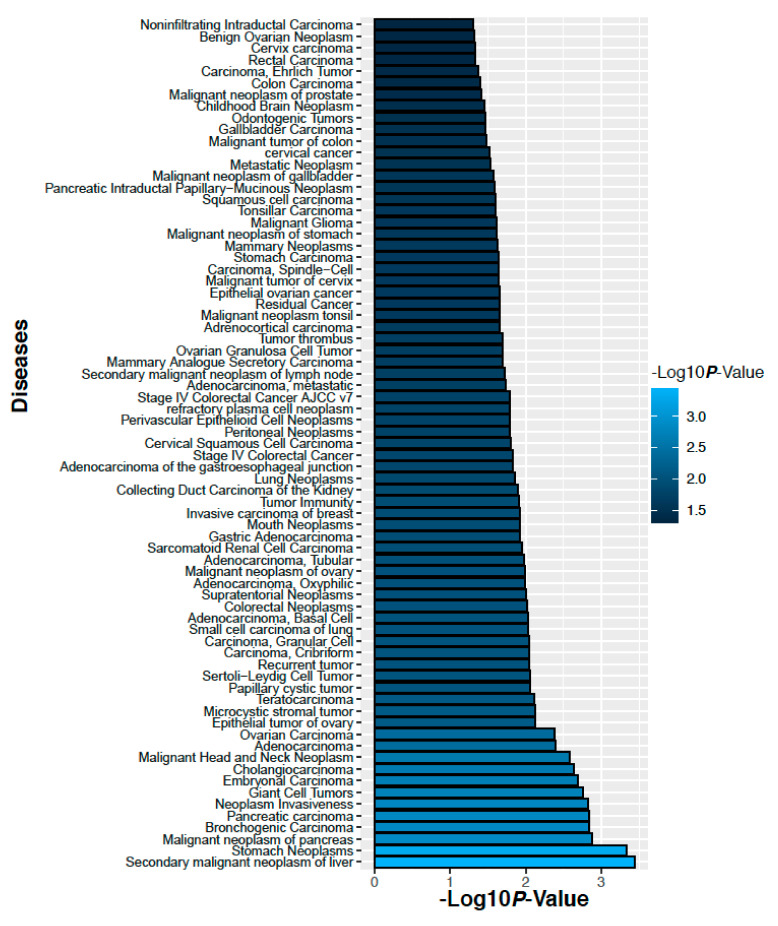
Diseases those are associate with the genes corresponding to the altered 14 proteins in the ovarian cancer.

**Figure 12:**
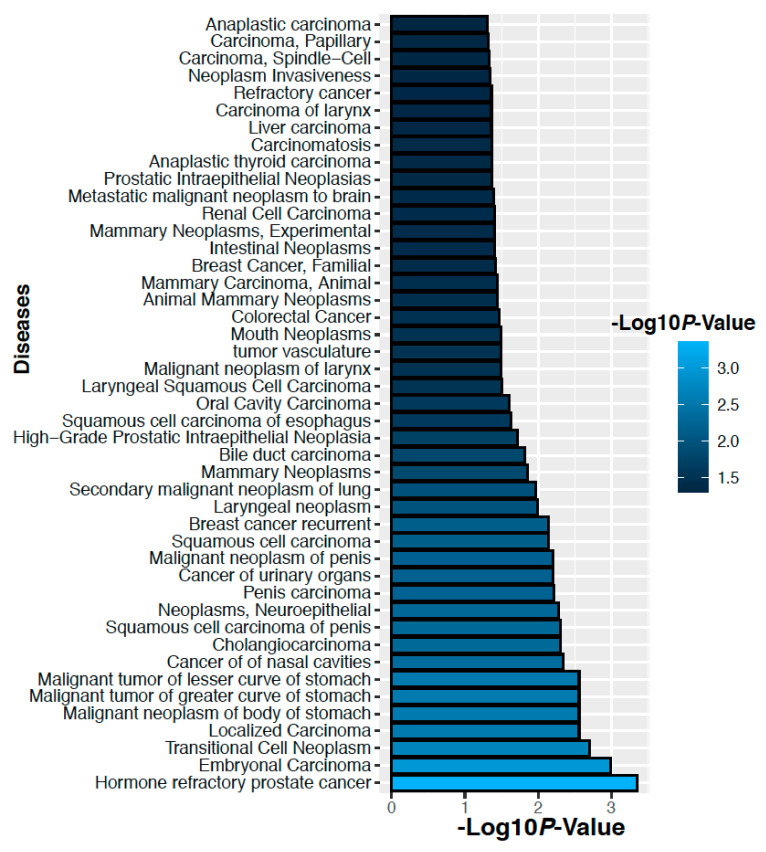
Diseases associated with the genes corresponding to the altered 3 proteins in colorectal cancer.

We have analyzed gene-disease association studies using the DisGeNET database that contains validated gene markers for each disease [22]. Carcinoma and neoplasm diseases associated with the genes corresponding to the altered 14 proteins in the ovarian cancer and their significance levels are shown in Figure 11. Similarly, carcinoma and neoplasm diseases associated with the genes corresponding to the altered 3 proteins in colorectal cancer and their significance level are shown in Figure 12. We observed that many cancers associated with our identified proteins.

### 2.8. In Vivo Toxicological Study

Twenty days toxicity study on swiss albino mice have shown that NH3 is less toxic compared to clinically used drug cisplatin at both lower (2.5 mg/kg) and higher doses (5 mg/kg). All mice were alive in control group and NH3-treated group at the lower dose, but only one mouse was alive in cisplatin-treated group till the end of the experiment at the same dose. The rest of the mice of cisplatin-treated group died within 15 days of experiment at the lower dose. Similarly, at the higher dose of cisplatin none of the mice was alive till the end of the experiment and most of them died within ten days of the experiment. However, all mice were alive in control group and only one mouse died following administration of NH3 at the higher dose. 

**Figure 13:**
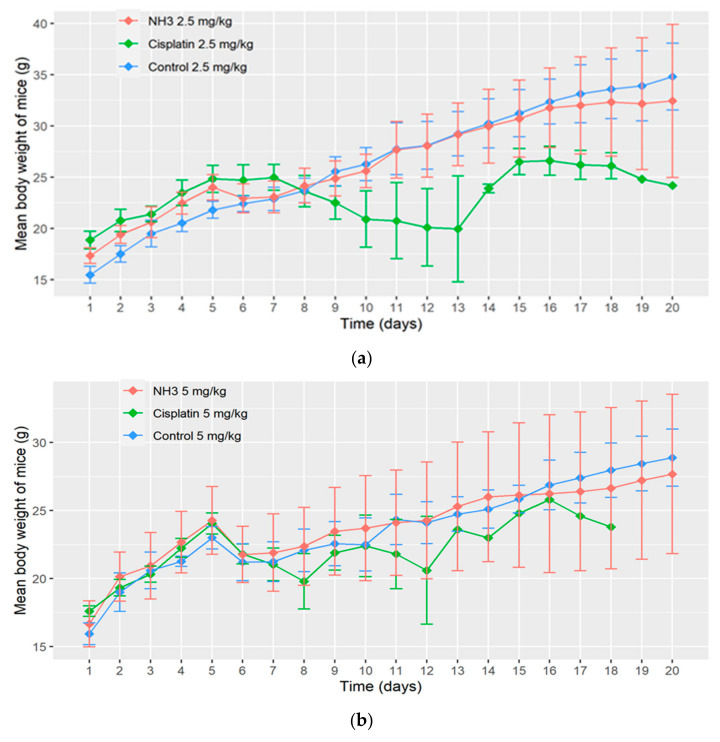
(**a**) Change in body weight of mice in different treatment groups at a dose of 2.5 mg/kg; (**b**) Change in body weight of mice in different treatment groups at a dose of 5 mg/kg.

The change in mean body weight of mice in different treatment groups is depicted in Figure 13, showing that body weight has been decreased drastically in cisplatin-treated group at both lower and higher doses. The implementation of repeated measures two-way analysis of variance showed that decrease in body weight is highly significant (*p*-value < 0.001) in cisplatin-treated groups at both doses compared to corresponding control groups. Decrease in body weight is also significant in NH3-treated groups compared to corresponding control groups. However, the intensity of the significance is less in this case. Observed *p*-values for paired groups are shown in Table 5.

Biochemical investigations for serum SGOT, SGPT and creatinine level have been presented in Table 6, which also indicates the fewer toxicity profile of NH3 compared to standard anticancer drug cisplatin.

### 2.9. Combination Study

Binary sequenced combinations of NH3 with curcumin and EGCG have been investigated in both ovarian and colorectal cancer models using four different cell lines. In terms of synergistic outcome, combination of NH3 with curcumin proved to be more beneficial than that with EGCG. In ovarian cancer models, the degree of synergism observed from combination of NH3 with curcumin is greater in parent A2780 cell line than in resistant A2780^cisR^ cell line. Degree of synergism is found to increase with the increase in added concentrations for 0/4 and 4/0 sequences of administration of NH3 in combination with curcumin against A2780^cisR^ cell line. However, converse is observed for bolus additions (Table 7). Taking together, combination of NH3 with curcumin at lower concentration is best for 0/4 sequence of administration followed by 4/0 sequence in regards to synergistic outcome against ovarian cancer model. 

While NH3 in combination with curcumin against HT-29 cell line has displayed synergism at all added concentrations for the 4/0 and bolus administrations. However, antagonism is observed at all concentrations for the 0/4 sequence of administration. Greater synergism is shown at lower concentrations than at higher added concentrations for the 4/0 sequence but the converse is true for the bolus administration in HT-29 cell line. Against Caco-2 cell line, synergistic effect is produced at ED_50_ level irrespective of the sequence of administration. However, at ED_75_ level, the synergism is found to decrease for all sequences of administration, and it is actually additive for 0/4 combination of NH3 with Cur in Caco-2 cell line (Table 8). With increase in concentration to ED_90_, all sequences of administration are found to display antagonism. 

Prospective benefits of synchronized addition of curcumin and cisplatin have been described against various cancers e.g., lymphoma [23], lung cancer [24,25] and bladder cancer in literature [26]. Curcumin in combination with platinum drugs (cisplatin, oxaliplatin) was reported to show sequence and dose-dependent synergism in ovarian cancer models in earlier studies from host laboratory as well [27]. Ulukaya et al. reported synergism obtained from combination of a tumor active palladium compound with curcumin [28]. The mechanism behind the synergistic outcome from combination of NH3 with curcumin could be associated with the role of the latter as a prooxidant [26,29,30]. Due to prooxidant activity, oxidative stress would be generated after entry of curcumin into the cells through dysregulation of gluthathione and glutathione-S- transferase levels. Oxidative stress would confer the cells ability to produce signals for MEK activation followed by ERK1/2 activation which would lead towards apoptotic and necrotic cell death [26]. Additionally, depletion of glutathione and GSTs would cause the reactive species to interact with DNA in an efficient manner for maximum cell killing. The idea is supported from palladium-DNA binding study as well in HT-29 cell line where 3-folds increase in palladium-DNA binding level from combined treatment of NH3 with curcumin is observed than single treatment of NH3 (data not shown). Literature suggests that prooxidant activity of curcumin is predominant mainly at a lower concentration [31] which is also corroborated with the results from the present study where it has been observed that CI values are more synergistic at ED_50_ level than ED_75_ and ED_90_ levels. Moreover, generation of reactive species from metabolism of NH3 would cause oxidative stress from other side and would lead towards cell death using multiple signaling pathways.

NH3, when combined with EGCG, has demonstrated significant synergism only at ED_50_ for bolus administration against ovarian A2780 cancer cell line. However, moderate synergism is found at ED_90_ level for 0/4 and 4/0 sequences, whereas bolus addition has displayed antagonism (Table 7). Against A2780^cisR^ cell line, 0/4 sequence of administration has exhibited synergism irrespective of the sequence of administration and the degree of synergism is found to increase with the increase in concentration. Bolus addition of NH3 with EGCG has also produced synergism at ED_75_ and ED_90_ level but antagonism is found at ED_50_ level in cisplatin-resistant A2780^cisR^ cell line. However, 4/0 sequence of administration has displayed additiveness at all concentrations. In HT-29 colorectal cancer line, combination of NH3 with EGCG has shown concentration-dependent synergism. At ED_90_ level, strong synergism is evident but strong antagonism is seen at ED_50_ for all sequences of administration (Table 8). In other colorectal cancer cell line Caco-2, the combination of NH3 with EGCG has displayed synergism at ED_50_ and ED_75_ for 0/0 and 0/4 sequences of administration. In contrast, 4/0 sequence is seen to be additive to antagonistic irrespective of concentrations.

Current literature shows that EGCG inhibits cancer cells growth in vitro and in vivo synergistically in combination with ascorbic acid, curcumin, 6-gingerol, quercetin, sulforaphane, raphasatin, proanthocyanidins, and other natural small molecules [32]. Synergism from combination with chemotherapeutics is also evident through the ability of EGCG to sensitize cancer cells towards chemotherapeutic drugs such as cisplatin [33], bleomycin [34], docetaxel, capecitabine, paclitaxel [35], and doxorubicin. Sequence and dose-dependent synergism was also reported from the host laboratory from combination of EGCG with cisplatin and designed palladiums [36]. A number of molecular pathways have been linked with the antitumor activity of EGCG, through which it displays synergism in combination with other phytochemicals and chemotherapeutics [37,38].

## 3. Materials and Methods

### 3.1. Chemistry (Reagents and Chemicals)

Potassium tetrachloropalladate (K2[PdCl4]); 8-Hydroxyquinoline (Sigma Chemical Company, St. Louis, MO, USA); HCl (Ajax Chemicals, Auburn, Australia); ethanol (Merck Pty. Ltd., Kilsyth, Australia).

### 3.2. Synthesis of [Bis(1,8-quinolato)palladium (II)] Coded as NH3

0.5 millimole of potassium tetrachloropalladate (0.163 g) was dissolved in 7.5 mL of mQ water to which 0.25 mL of concentrated HCl was added. Five millimoles of 8-Hydroxyquinoline (0.726 g) dissolved in 7.5 mL of ethanol, was added drop wise over 1 h to the solution of potassium tetrachloropalladate at 40 °C. The reaction mixture was stirred at room temperature for 2 weeks. 4 mL of 0.25 M hydrochloric acid was added to the mixture and stirring was continued for 1 week at room temperature. The mixture was centrifuged at 5500 rpm for 10 min to collect precipitate of NH3. The crude product was purified by dissolving in 0.05 M HCl, followed by filtration and collected after washing successively with ice-cold water and ethanol. The purified product was air dried and weighed. Vapour diffusion technique was used during production of suitable crystals of NH3 for crystallography using methanol and diethyl ether as solvents.

### 3.3. Elemental and Spectral Characterization

Elemental microanalysis of the designed compound for C, H, and N was determined using microanalysis facility available at the Macquarie University. Model PE2400 CHNS/O (PerkinElmer, Shelton, CT, USA) analyzer was used for the determination of C, H, and N. Palladium content was determined by graphite furnace atomic absorption spectroscopy (AAS) available at University of Sydney. IR spectrum of NH3 was recorded using a PerkinElmer FT-IR spectrometer. To obtain mass spectra, solution of NH3 was made in 10% DMF and 90% methanol and then 2 µL was transferred into a drawn glass capillary pre sputter coated with silver and inserted into a nanospray holder attached to a Thermo Orbitrap mass spectrometer available at the Bioanalytical mass spectrometry facility at the University of New South Wales. The 1H NMR spectrum of NH3 (dissolved in deuterated DMSO) were recorded on a Bruker DPX400 spectrometer using a 5 mm high-precision Wilmad NMR tube at 300 K (±1 K).

NH3 Yield: 0.163 g (82.32%). Anal. Calcd. for [PdC_18_H_12_N_2_O_2_] (394.73 g/mol): C = 54.77%, H = 3.06%, N = 7.10%, Pd = 26.96%. Found: C = 55.15%, H = 3.67%, N = 6.94%, Pd = 26.64%. Selected IR data (KBR, cm^−1^): ῦ = 3055, 2360, 2341, 1572, 1497, 1461, 1372, 1316, 1283, 1214, 1172, 1114, 824, 738, 657, 529. ^1^H NMR (400 MHz, D_2_O): δ =8.58 (d, due to C_2_H); 8.47 (d, due to C_2_H); 7.67 (q, due to C_6_H); 7.46 (t, due to C_3_H); 7.11 (d, due to C_4_H); 6.93 (d, due to C_7_H); 3.69 (s, due to water); 2.49 (s, due to DMSO). MS (ESI) m/z (%): 395.00 (100) = [PdC_18_H_12_N_2_O_2_], 789.99 (18) = [PdC_18_H_12_N_2_O_2_ + PdC_18_H_12_N_2_O_2_], 811.97 (33) = [PdC_18_H_12_N_2_O_2_ + PdC_18_H_12_N_2_O_2_ + H_2_O + 4H], 827.71 (7) = [PdC_18_H_12_N_2_O_2_ + PdC_18_H_12_N_2_O_2_ + 2H_2_O + 2H], 416.98 (18) = [PdC_18_H_12_N_2_O_2_ + H_2_O + 3H], 445.35 (13) = [PdC_18_H_12_N_2_O_2_ + Cl + H_2_O − 3H]

### 3.4. Biological Activity

Antitumor activity and proteomics: Cytotoxicity of NH3 against seven cancer cell lines along with that for cisplatin (used as reference) was determined using the MTT reduction assay [39,40]. The method for determining single drug cytotoxicity and activity of drugs in sequenced combination was the same as described in our previous articles [8]. In brief, different concentrations of drugs (cisplatin ranging from either 0.8 to 100 µM or 0.16 to 20 µM and NH3 ranging from 0.008 to 1 µM) obtained through serial dilution were added to the cells contained in 96 well. The plates were then left to incubate under normal growth conditions for 72 h. Each treatment was done in triplicate in the same plate, while the control wells did not receive any drug treatment (contained cells and medium only). Four hours after the addition of the MTT solution (50 µL per well of 1 mg/mL MTT solution), the purple formazan crystals produced from the reduction of MTT were dissolved in 150 µL DMSO and read with a iMarkTM Bio-Rad Microplate Reader Version 1.04.02.E. The IC_50_ values were obtained from the results of at least three independent experiments. Proteomic study was conducted following same procedure mentioned before in our published research article [9].

### 3.5. Toxicity Study

Experimental animals: Thirty six Swiss-albino adult male mice (body weight range 20 ± 5 gm) were purchased from the Department of Pharmacy, Jahangirnagar University, Bangladesh. Mice were housed in a 12 h day night cycle (light on at 7 am and off at 7 pm) providing bench light of 350 lux, at 25 ± 2 °C temperature and 55 ± 10% humidity. Mice were reared in the cages (580 × 375 × 190 mm), supplied with commercially available food pellet [Rodent diet, ICDDRB, Bangladesh] and drinking water ad libitum. Body weight of each mouse was measured twice every day and other physical changes recorded.

Animal preparation, drug administration and treatment: After 5 days of acclimation, mice were randomly divided into six groups: control 1 (C1, receiving the solvent DMSO at 2.5 mg/kg body weight), control 2 (C2, receiving the solvent DMSO at 5 mg/kg body weight), experimental group1 (E1, receiving the investigational drug NH3 at a dose of 2.5 mg/kg body weight), experimental group 2(E2, receiving the investigational drug NH3 at a dose of 5 mg/kg body weight), standard1 (S1, receiving the standard anticancer drug cisplatin at a dose of 2.5 mg/kg body weight) and standard 2 (S2, receiving the standard anticancer drug cisplatin at a dose of 5 mg/kg body weight), respectively. All the drugs and control vehicles had been administered subcutaneously.

Animal sacrifice, blood and organs collection: Twenty four hours following the last treatment and test, the mice were kept in fasting overnight. Then, they were anaesthetized with intraperitoneal injection of sodium pentobarbital (35 mg/kbw) [Phenobarbital, Incepta Pharmaceuticals, Bangladesh] and sacrificed as shown in Table 9

Blood was collected immediately, centrifuged at 1000 rpm, collected plasma and serum were preserved at –80 °C for biochemical study (SGPT, SGOT and Creatinine). Biochemical investigations were carried out in an auto analyzer (Photometer 5010 V5+, Robert Riely, Berlin, Germany) using Sigma Aldrich (Saint Louis, MO, USA) reagent kit. The mice model preliminary toxicity study was approved by the Biosafety, Biosecurity and Ethical Committee [Approval Number: BBEC-JU/M2019(12)1] of Jahangirnagar University, Savar, Dhaka, Bangladesh. Statistical analysis was conducted through SPSS using Satterthwaite’s method for equality of variances.

## 4. Conclusions

Palladium complex [Bis(1,8-quinolato)palladium (II)] coded as NH3 has been synthesized and characterized, followed by studies on its activity alone and in combination with ovarian and colorectal cancer cell lines. Proteomic studies were carried out to determine key proteins associated with antitumor activity of NH3. Theoretical studies have also been carried out on protein–protein interaction.

## Figures and Tables

**Table 1:** Comparison of crystallographic unit cell constants in P2_1_/c.

Structure	*a*	*b*	*c*	*β*	Volume (Å^3^)	Z	Temp (Kelvin)
HQUIPD	11.49	4.77	15.31	121.9	712.4	2	293
HQUIPD01	11.216	4.719	14.993	120.22	685.7	2	100
NH3	9.340	10.110	14.844	100.77	1385.6	4	150

**Table 2:** Protein spots that underwent significant changes in expression after treatment with NH3 alone in A2780 cell line and their identification (name, mass, Da/pI, mascot score, matched peptides, percentage of sequence coverage).

Spot No	Change in Expression	Fold Change	Protein Name	Mass (Da)/pI	Mascot Score	No of Matched Peptides	Sequence Coverage (%)
6	Downregulated	4.8	Actin, cytoplasmic 1	41,710/5.29	520	16	30
12	Downregulated	1.63	Vimentin	53,619/5.06	739	6	45
13	Downregulated	3.23	60 kDa heat shock protein, mitochondrial	61,016/5.70	518	19	24
18	Downregulated	4.56	Endoplasmin	92,411/4.76	647	36	21
45	Downregulated	2.00	78 kDa glucose-regulated protein	72,288/5.07	820	28	27
118	Downregulated	11.39	Polyubiquitin-B	25,746	40	Not given	Not given
155	Downregulated	6.63	Histone H3.3	15,319/11.27	120	11	27

**Table 3:** Protein spots that underwent significant changes in expression after treatment with NH3 alone in A2780^cisR^ cell line and their identification (name, mass, Da/pI, mascot score, matched peptides, percentage of sequence coverage).

Spot No	Change in Expression	Fold change	Protein Name	Mass (Da)/pI	Mascot Score	No of Matched Peptides	Sequence Coverage (%)
1	Downregulated	1.51	Actin, cytoplasmic 1	41,710/5.29	520	16	30
Cn9	Downregulated	2.98	40S ribosomal protein SA	32,833/4.79	130	10	17
Cn16	Downregulated	1.69	Heat shock cognate 71 kDa protein	70,854/5.37	686	27	21
Cn23	Upregulated	2.47	Keratin, type II cytoskeletal 1	65,999	31	Not given	Not given
Cn34	Downregulated	7.86	Elongation factor Tu, mitochondrial	49,510/7.26	88	13	16
Cn41	Downregulated	2.93	Citrate synthase, mitochondrial	51,680/8.45	97	14	16
Cn56	Downregulated	3.87	Transitional endoplasmic reticulum ATPase	89,266/5.14	208	14	10
Cn69	Downregulated	2.27	Cofilin-1	18,491/8.22	144	10	22
Cn79	Downregulated	2.79	Actin, cytoplasmic 1	41,710/5.29	231	19	48

**Table 4:** Protein spots that underwent significant changes in expression after treatment with NH3 alone in HT-29 cell line and their identification (name, mass, Da/pI, mascot score, matched peptides, percentage of sequence coverage).

Spot No	Change in Expression	Fold Change	Protein Name	Mass (Da)/pI	Mascot Score	No of Matched Peptides	Sequence Coverage (%)
Hn70	Downregulated	2.14	Nucleoside Diphosphate kinase B	17,287/8.52	308	5(4)	71
Hn89	Downregulated	1.85	Annexin A1	38,690/6.57	166	25	54
Hn119	Downregulated	1.91	Histone H4	11,360/11.36	58	6	29

**Table 5:** Observed *p*-values from Satterthwaite’s method for equality of variances.

Tested Group	*p*-Value
Control vs cisplatin (2.5 mg/kg)	2.2 × 10^−16^ ***
Control vs NH3 (2.5 mg/kg)	1.043 × 10^−5^ ***
Control vs cisplatin (5 mg/kg)	1.584 × 10^−5^ ***
Control vs NH3 (5 mg/kg)	0.03752 **

*** highly significant at 5% level of significance; ** significant at 5% level of significance.

**Table 6:** Biochemical study parameters.

Mice Group	SGOT (IU/L)	SGPT (IU/L)	Creatinine (mg/dL)
C1 (Control 2.5 mg/kg)	48 ± 7	42 ± 6	0.6 ± 0.1
C2 (Control 5 mg/kg)	47 ± 3	41 ± 3	0.6 ± 0.2
S1 (Cisplatin 2.5 mg/kg)	57 ± 4	61 ± 5	0.7 ± 0.3
S2 (Cisplatin 5 mg/kg)	61 ± 2	59 ± 7	0.9 ± 0.2
E1 (NH3 2.5 mg/kg)	53 ± 4	45 ± 3	0.9 ± 0.2
E2 (NH3 5 mg/kg)	59 ± 5	48 ± 4	0.7 ± 0.2

**Table 7:** CI values (at ED_50_, ED_75_, ED_90_) and dose-effect parameters (median effect dose Dm, the exponent defining the shape of the dose–effect curve m, correlation coefficient r) applying to combinations of NH3 with curcumin and EGCG in the A2780 and A2780^cisR^ cell line.

Cell Line	Drug or Drug Combination	Sequence (h)	Molar Ratio	CI Values at					
				ED_50_	ED_75_	ED_90_	D_m_	m	r
A2780	NH3		1:63.55	N/A	N/A	N/A	0.18	1.97	0.94
	Curcumin			N/A	N/A	N/A	8.63	1.25	1.00
	NH3 + Curcumin	0/0		0.59	0.92	1.48	0.05	0.93	1.00
	NH3 + Curcumin	0/4		0.30	0.71	1.74	0.02	0.69	0.99
	NH3 + Curcumin	4/0		0.46	0.80	1.42	0.04	0.86	1.00
	NH3		1:64.2	N/A	N/A	N/A	0.18	1.97	0.94
	EGCG			N/A	N/A	N/A	10.34	0.94	0.95
	NH3 + EGCG	0/0		0.44	0.89	1.97	0.04	0.71	0.94
	NH3 + EGCG	0/4		1.22	0.93	0.77	0.10	1.95	0.97
	NH3 + EGCG	4/0		1.34	1.02	0.84	0.11	1.97	0.99
A2780^cisR^	NH3		1:76.07	N/A	N/A	N/A	0.23	2.05	0.99
	Curcumin			N/A	N/A	N/A	16.74	1.56	1.00
	NH3 + Curcumin	0/0		0.83	0.77	0.72	0.09	2.02	0.94
	NH3 + Curcumin	0/4		0.55	0.66	0.80	0.06	1.37	0.97
	NH3 + Curcumin	4/0		0.86	0.81	0.76	0.10	1.99	0.94
	NH3		1:64.2	N/A	N/A	N/A	0.23	2.05	0.99
	EGCG			N/A	N/A	N/A	8.90	0.93	0.95
	NH3 + EGCG	0/0		1.15	0.85	0.69	0.11	1.99	0.97
	NH3 + EGCG	0/4		0.91	0.67	0.55	0.09	1.99	0.92
	NH3 + EGCG	4/0		0.95	1.01	1.20	0.09	1.19	0.98

**Table 8:** CI values (at ED_50_, ED_75_, ED_90_) and dose-effect parameters (median effect dose Dm, the exponent defining the shape of the dose–effect curve m, correlation coefficient r) applying to combinations of NH3 with curcumin and EGCG in the HT-29 and Caco-2 cell line.

Cell Line	Drug or Drug Combination	Sequence (h)	Molar Ratio	CI Values at					
				ED_50_	ED_75_	ED_90_	D_m_	m	r
HT-29	NH3		1:199.97	N/A	N/A	N/A	0.38	2.06	0.98
	Curcumin			N/A	N/A	N/A	16.63	1.25	0.97
	NH3 + Curcumin	0/0		0.98	0.86	0.77	0.07	1.62	1.00
	NH3 + Curcumin	0/4		1.21	1.29	1.40	0.08	1.27	0.99
	NH3 + Curcumin	4/0		0.50	0.57	0.68	0.03	1.16	1.00
	NH3		1:278.26	N/A	N/A	N/A	0.38	2.06	0.98
	EGCG			N/A	N/A	N/A	3.67	0.39	0.96
	NH3 + EGCG	0/0		5.69	1.02	0.46	0.07	1.29	0.96
	NH3 + EGCG	0/4		9.00	1.33	0.50	0.11	1.67	1.00
	NH3 + EGCG	4/0		7.54	1.24	0.52	0.10	1.43	1.00
Caco-2	NH3		1:61.25	N/A	N/A	N/A	0.56	1.96	0.93
	Curcumin			N/A	N/A	N/A	21.90	1.08	1.00
	NH3 + Curcumin	0/0		0.38	0.65	1.19	0.08	0.81	1.00
	NH3 + Curcumin	0/4		0.89	1.06	1.33	0.19	1.11	1.00
	NH3 + Curcumin	4/0		0.71	0.93	1.28	0.15	1.01	1.00
	NH3		1:177.17	N/A	N/A	N/A	0.56	1.96	0.93
	EGCG			N/A	N/A	N/A	107.58	1.32	0.94
	NH3 + EGCG	0/0		0.45	0.69	1.09	0.13	0.98	0.95
	NH3 + EGCG	0/4		0.34	0.73	1.58	0.10	0.77	0.99
	NH3 + EGCG	4/0		0.90	1.15	1.49	0.26	1.19	0.98

**Table 9:** Activity during in vivo toxicological study.

Activity	Duration/Date During Experiment
Acclimation of the animals	5 days
Treatment phase 1	6th to 10th day
Relax phase	11th to 15th day
Treatment phase 2	16th to 20th day
Treatment off/Preparation for sacrifice	21st day
Sacrifice of animals	22nd day

## Data Availability

Not applicable.

## Supplementary Materials

The following are available online at https://www.mdpi.com/article/10.3390/ijms22168471/s1.
