# Supplementary material for: Promising Anticancer Activity of [Bis(1,8-quinolato)palladium (II)] Alone and in Combination
Source: Int J Mol Sci. 2021 Aug 6;22(16):8471. doi: 10.3390/ijms22168471 (PMC8395214; doi:10.3390/ijms22168471)

Table S1. Crystallographic details for NH3

Refinement model formula	$C_{18}H_{12}N_2O_2Pd$
Model molecular weight	394.70
Crystal system	Monoclinic
Space group	$P2_1$/c(#14)
a	9.3997(4) Å
b	10.1091(4) Å
c	14.8435(6) Å
β	100.769(4)º
V	1385.62(10) Å^3
D_c	1.892 g cm^{-3}
Z	4
Crystal Size	0.062x0.060x0.016 mm
Crystal Colour	Orange
Crystal Habit	Plate
Temperature	150(1) Kelvin
λ(Cu Kα)	1.5418 Å
μ(Cu Kα)	10.906 mm^{-1}
$T_{min,max}$	0.54, 1.00
$2\theta_{max}$	152.46º
hkl range	-11 8, -12 12, -18 18
N	14466
N_{ind}	2895(R_{merge} 0.0884)
N_{obs}	2155(I > 2σ(I))

N_{var}	209
Residuals* $R1(F)$, $wR2(F^2)$	0.0470, 0.1135
GoF(all)	1.204
Residual Extrema	-0.917, 1.786 $e^- Å^{-3}$

Table S2. Selected non-hydrogen bond lengths (Å)

First Atom	Second atom	Distance	First atom	Second atom	Distance
Pd(1)	N(1)	2.003(5)	Pd(1)	O(2)	2.002(4)
Pd(1)	N(2)	2.001(4)	Pd(1)	O(1)	2.007(4)
O(1)	C(1)	1.317(7)	O(2)	C(10)	1.324(7)
N(1)	C(3)	1.301(8)	N(1)	C(2)	1.388(7)
N(2)	C(12)	1.312(8)	N(2)	C(11)	1.380(7)
C(1)	C(9)	1.391(8)	C(1)	C(2)	1.421(8)
C(2)	C(6)	1.419(8)	C(3)	C(4)	1.408(9)
C(4)	C(5)	1.375(9)	C(5)	C(6)	1.403(9)
C(6)	C(7)	1.420(8)	C(7)	C(8)	1.367(9)
C(8)	C(9)	1.415(9)	C(10)	C(18)	1.389(8)
C(10)	C(11)	1.437(8)	C(11)	C(15)	1.401(8)
C(10)	C(11)	1.437(8)	C(11)	C(15)	1.401(8)
C(12)	C(13)	1.401(8)	C(13)	C(14)	1.373(8)
C(14)	C(15)	1.423(8)	C(15)	C(16)	1.416(8)
C(16)	C(17)	1.364(9)	C(17)	C(18)	1.410(9)

Table S3. Selected non-hydrogen bond angles (°)

First atom	Second atom	Third atom	Angle
N(1)	Pd(1)	O(2)	96.05(18)
N(1)	Pd(1)	N(2)	179.6(2)
O(2)	Pd(1)	N(2)	84.07(18)
N(1)	Pd(1)	O(1)	84.03(19)
O(2)	Pd(1)	O(1)	178.92(18)
N(2)	Pd(1)	O(1)	95.84(18)
C(1)	O(1)	Pd(1)	110.6(4)
C(10)	O(2)	Pd(1)	110.7(3)
C(3)	N(1)	C(2)	120.6(5)
C(3)	N(1)	Pd(1)	129.6(4)
C(2)	N(1)	Pd(1)	109.7(4)
C(12)	N(2)	C(11)	120.0(5)
C(12)	N(2)	Pd(1)	129.6(4)
C(11)	N(2)	Pd(1)	110.4(4)
O(1)	C(1)	C(9)	123.9(6)
O(1)	C(1)	C(2)	119.4(5)
C(9)	C(1)	C(2)	116.7(5)
N(1)	C(2)	C(6)	120.6(5)
N(1)	C(2)	C(1)	116.2(5)
C(6)	C(2)	C(1)	123.2(5)
N(1)	C(3)	C(4)	122.1(5)
C(5)	C(4)	C(3)	118.6(5)
C(4)	C(5)	C(6)	121.1(5)
C(5)	C(6)	C(2)	116.9(5)
C(5)	C(6)	C(7)	125.7(6)
C(2)	C(6)	C(7)	117.4(6)
C(8)	C(7)	C(6)	120.0(6)
C(7)	C(8)	C(9)	121.8(5)
C(1)	C(9)	C(8)	121.0(5)
O(2)	C(10)	C(18)	124.3(5)
O(2)	C(10)	C(11)	119.1(5)
C(18)	C(10)	C(11)	116.6(5)
N(2)	C(11)	C(15)	121.6(5)
N(2)	C(11)	C(10)	115.7(5)
C(15)	C(11)	C(10)	122.7(5)
N(2)	C(12)	C(13)	122.0(5)
C(14)	C(13)	C(12)	119.5(5)
C(13)	C(14)	C(15)	119.8(5)
C(11)	C(15)	C(16)	118.4(5)
C(11)	C(15)	C(14)	117.1(5)
C(16)	C(15)	C(14)	124.5(6)
C(17)	C(16)	C(15)	119.2(6)
C(16)	C(17)	C(18)	122.7(6)
C(10)	C(18)	C(17)	120.4(5)

Supplementary Figure S1. Dose response curves in A2780 cell line

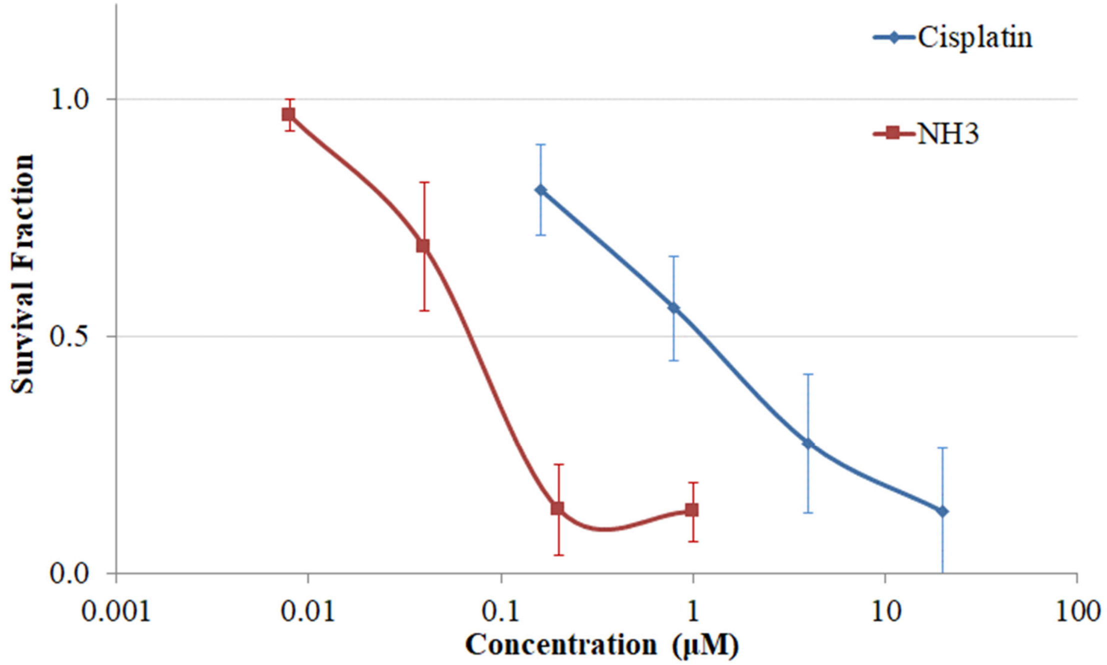

Supplementary Figure S2. Dose response curves in A2780^{cisR} cell line

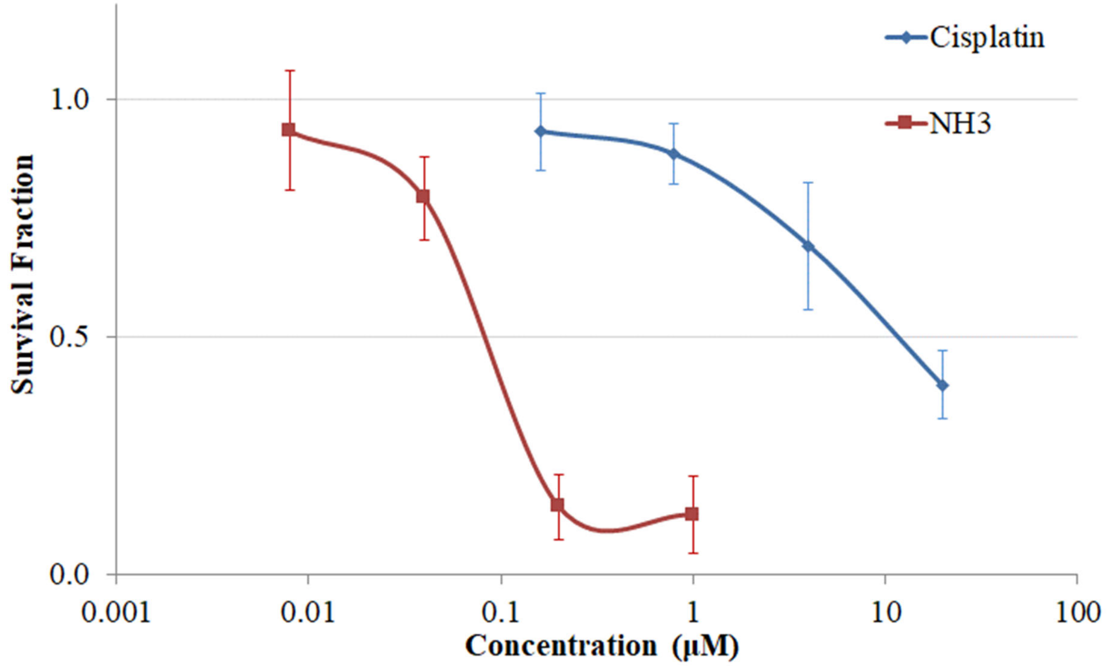

Supplementary Figure S3. Dose response curves in HT-29 cell line

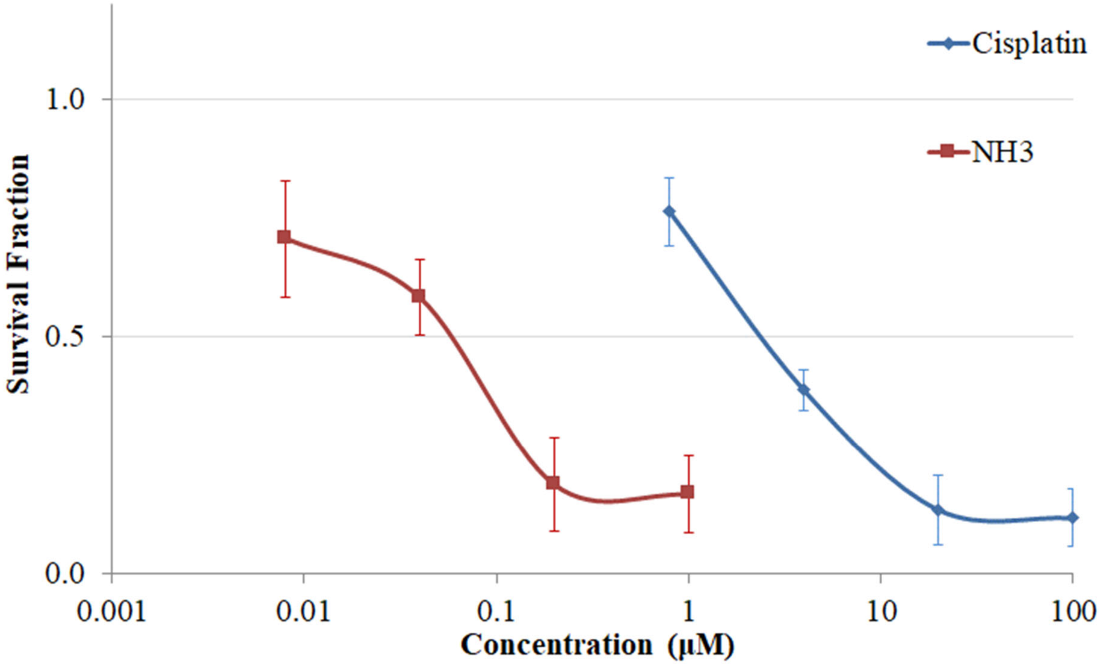

Supplementary Figure S4. Dose response curves in Caco-2 cell line

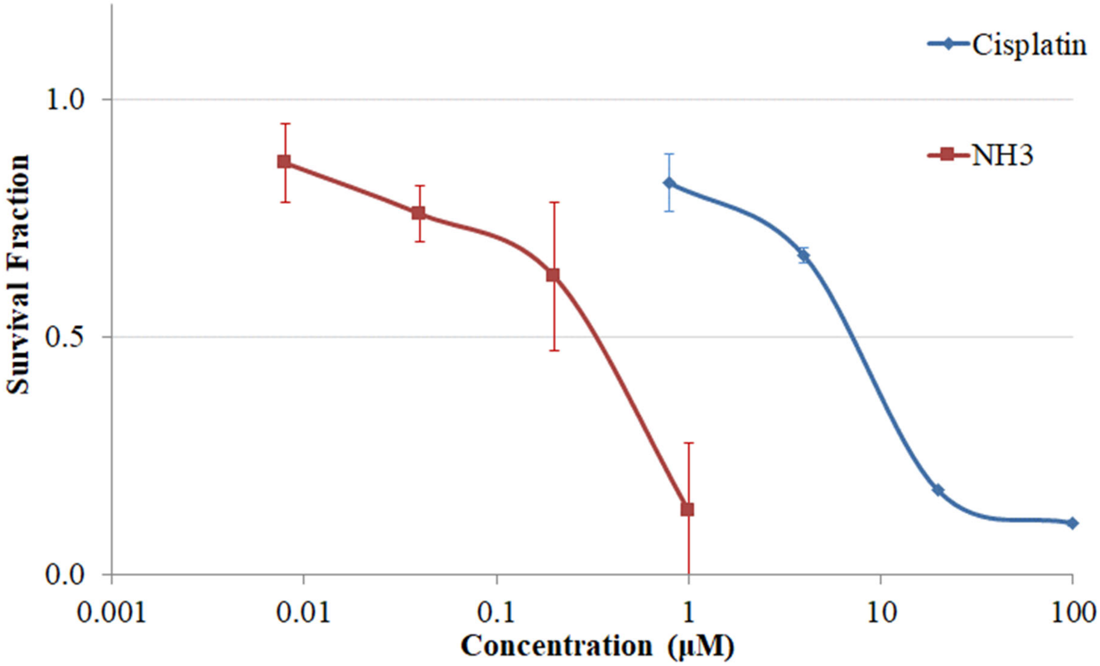

Supplementary Figure S5. Dose response curves in A2780^{ZDO473R}, HeLa and MCF-7 cell line

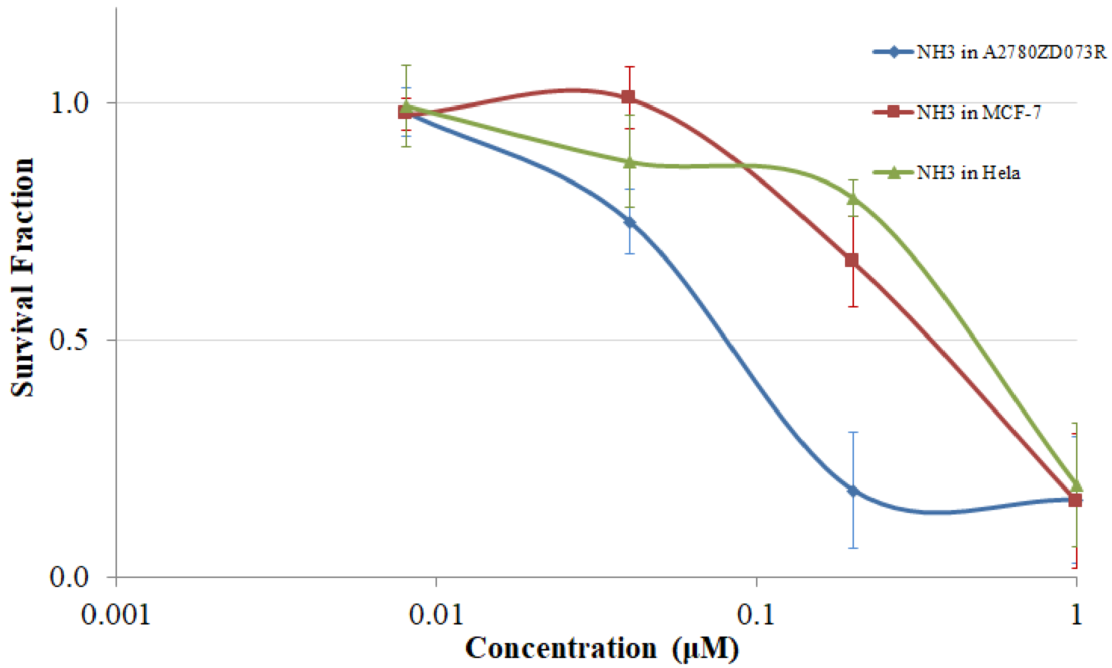

Supplement: Supplementary file 1 [file ijms-22-08471-s001.zip › ijms-1310304-supplementary.pdf]
